# Psittacosis caused severe community-acquired pneumonia accompanied by acute hypoxic respiratory failure: a multicenter retrospective cohort study from China

**DOI:** 10.1186/s12879-023-08283-z

**Published:** 2023-08-14

**Authors:** Xiao Tang, Na Wang, Gang Liu, Hai Tan, Ai-Min Li, Yan-Qiu Gao, Meng-Ying Yao, Na Wang, Hui-Dan Jing, Qing-Guo Di, Liang Chen, Rui Wang, Xu-Yan Li, Ying Li, Xue Yuan, Yu Zhao, Qi Li, Zhao-Hui Tong, Bing Sun

**Affiliations:** 1 Department of Respiratory and Critical Care Medicine, Beijing Institute of Respiratory Medicine and Beijing Chao-Yang Hospital, Capital Medical University, Beijing, China; 2 Department of Pulmonary and critical care medicine, Beijing Luhe Hospital, Capital Medical University, Beijing, China; 3 Department Pulmonary and critical care medical center, Xinqiao hospital, Army Medical University, the Chinese People’s Liberation Army Respiratory Disease Institute, Chongqing, China; 4 Department of Respiratory and Critical Care Medicine, General Hospital of Ningxia Medical University, Ningxia Hui Autonomous Region, Xi Ning, China; 5 Respiratory and Critical Care Medicine, First Hospital of Shanxi Medical University, Taiyuan, Shanxi Province China; 6 Respiratory Intensive Care Unit, Zhengzhou Central Hospital Affiliated to Zhengzhou University, Zhengzhou, Henan Province China; 7 Department of Respiratory Intensive Care Unit, The First Affiliated Hospital of Zhengzhou University, Zhengzhou, Henan Province China; 8 Department of Pulmonary, The first hospital of Fangshan district, Beijing, China; 9 Department of Intensive Care Unit, Daping Hospital, Army Medical University, Chongqing, China; 10 Department of Pulmonary and Critical Care Medicine, Cangzhou Central Hospital, Cangzhou, Hebei Province China; 11 Department of Respiratory and Critical Care Medicine, Beijing Jingmei Group General Hospital, Beijing, China; 12 Department of Respiratory and Critical Care Medicine, Beijing Institute of Respiratory Medicine and Beijing Chao-Yang Hospital, Capital Medical University, Beijing, China

**Keywords:** Psittacosis, Severe community-acquired pneumonia, Acute hypoxic respiratory failure, Fluoroquinolone, Intensive care unit

## Abstract

**Introduction:**

Psittacosis can cause severe community-acquired pneumonia (CAP). The clinical manifestations of psittacosis range from subclinical to fulminant psittacosis with multi-organ failure. It is essential to summarize the clinical characteristic of patients with severe psittacosis accompanied by acute hypoxic respiratory failure (AHRF).

**Methods:**

This retrospective study included patients with severe psittacosis caused CAP accompanied by AHRF from 19 tertiary hospitals of China. We recorded the clinical data, antimicrobial therapy, respiratory support, complications, and outcomes. *Chlamydia psittaci* was detected on the basis of metagenomic next-generation sequencing performed on bronchoalveolar lavage fluid samples. Patient outcomes were compared between the treatment methods.

**Results:**

This study included 45 patients with severe CAP and AHRF caused by psittacosis from April 2018 to May 2021. The highest incidence of these infections was between September and April. There was a history of poultry contact in 64.4% of the patients. The median PaO_2_/FiO_2_ of the patients was 119.8 (interquartile range, 73.2 to 183.6) mmHg. Four of 45 patients (8.9%) died in the ICU, and the median ICU duration was 12 days (interquartile range, 8 to 21) days. There were no significant differences between patients treated with fluoroquinolone initially and continued after the diagnosis, fluoroquinolone initially followed by tetracycline, and fluoroquinolone combined with tetracycline.

**Conclusion:**

Psittacosis caused severe CAP seems not rare, especially in the patients with the history of exposure to poultry or birds. Empirical treatment that covers atypical pathogens may benefit such patients, which fluoroquinolones might be considered as an alternative.

## Introduction

Psittacosis is caused by *Chlamydia psittaci*, an intracellular gram-negative bacterium. It is a zoonosis that commonly infects birds. Although not always present, exposure to birds is a major risk factor for infection [1]. Psittacosis was found to be responsible for 1.03% of all community-acquired pneumonias (CAPs) in a meta-analysis [2]. It also accounts for 2.3% of cases of severe CAP [3]. The clinical manifestations range from subclinical or brief to multi-organ failure that was less commonly reported as fulminant psittacosis [4–7]. Before the advent of antimicrobial agents, the mortality of pneumonia caused by *C. psittaci* was 15–20%[8]. However, mortality is rare today.

Psittacosis can be diagnosed on the basis of clinical presentation and tests that detect the human pathogenic *C. psittaci*, such as microimmunofluorescence, indirect fluorescence antibody, culture, or polymerase chain reaction (PCR)[1]. Metagenomic next-generation sequencing (mNGS) was recently developed for disease screening and diagnosis. Many recent reports have described the use of mNGS for the diagnosis of psittacosis in patients with severe CAP [9–11]. However, psittacosis is not routinely tested for in pneumonia diagnostic panels in many countries. Therefore, psittacosis may be underestimated and unrecognized, especially for the critically ill patients. Early diagnosis and treatment is essential for such patients. In this multicenter retrospective study, we evaluated the clinical characteristics and outcomes of patients with severe CAP and acute hypoxic respiratory failure (AHRF) caused by psittacosis admitted to the intensive care units (ICUs).

## Materials and methods

### Study design and patients

This retrospective study included patients with severe CAP and AHRF caused by psittacosis who were admitted to the 19 tertiary hospitals of China from April 2018 to May 2021. Patients were included if they fulfilled the following criteria: (1) severe CAP [12]; (2) AHRF (arterial partial pressure of oxygen [PaO_2_] < 60 mmHg on room air, and arterial partial pressure of carbon dioxide [PaCO_2_] < 45 mmHg), or need for > 6 L/min oxygen for respiratory support, and respiratory symptoms < 72 h; (3) *C. psittaci* detected in sputum or bronchoalveolar lavage fluid (BALF) using mNGS; (4) samples included blood, sputum, and BALF culture with negative results of routine microbiological tests; (5) ICU admission; (6)

the diagnosis of psittacosis pneumonia independently decided by two physicians according to the clinical manifestation, microbiological tests results and lung computed tomography (CT). This study was reviewed and approved by the Ethics Committee of Beijing Chao-Yang Hospital of China (2021-Ke-389). Because this was a retrospective study, consent was waived by the Ethics Committee of the Beijing Chao-Yang Hospital. All methods were carried out in accordance with relevant guidelines and regulations.

### Data collection

Demographic and clinical data of the patients were entered into an electronic case report form. The collected data included the demographic characteristics, comorbidities, symptoms, signs, laboratory tests, microbiological findings, and radiologic images of the lung (chest X-ray and computed tomography [CT]). The treatment process during ICU admission such as, antimicrobial therapy, respiratory support, complications, and outcomes were also recorded, in addition to an experienced radiologist in pulmonary imaging experience’s interpretation who was blinded to the clinical data reviewed the CT images. CT images were evaluated and defined according to the Fleischner Society glossary of terms for thoracic imaging [13]. The extent of disease at CT was evaluated as CT score [14].

### Microbiological tests

Sputum, blood, serum, and BALF samples were collected at admission and during ICU admission. Sputum and BALF samples were tested on bacterial and fungal smear and culture, and real-time PCR for common viruses that cause respiratory disease including influenza virus, adenovirus, rhinovirus, respiratory syncytial virus, cytomegalovirusm, etc. Serum samples were used for *M. pneumoniae, C. pneumonia*, and *L. pneumophila* antibodies.

BALF samples were processed using mNGS to screen for pathogenic microorganisms at Vision Medicals Co., Ltd. (Guangzhou, China). The BALF samples were then subjected to nucleic acid extraction (Vision Medicals Cat# VM001, Guangzhou, China). DNA libraries were prepared and sequenced on an Illumina Nextseq sequencer for clinical metagenomic analysis. Sequence analysis was performed through Vision Medicals’ IDseqTM commercial bioinformatic pipeline. Briefly, reads that mapped to human genome and plasmids were removed. And the remaining reads were taxonomically classified by aligning to Vision Medicals’ curated microbial database.

### Statistical analysis

SPSS software (version 22.0; IBM Corp., Armonk, NY, USA) was used for the statistical analysis. Continuous variables are presented as mean ± standard deviation (SD) or median (interquartile range, IQR). Chi-squared tests were used to analyze the categorical variables, and Mann–Whitney *U* test was used to analyze the continuous. Univariate analysis was used for the comparison of different treatments. *P-*value < 0.05 was considered to be statistically significant.

## Results

**Fig. 1:**
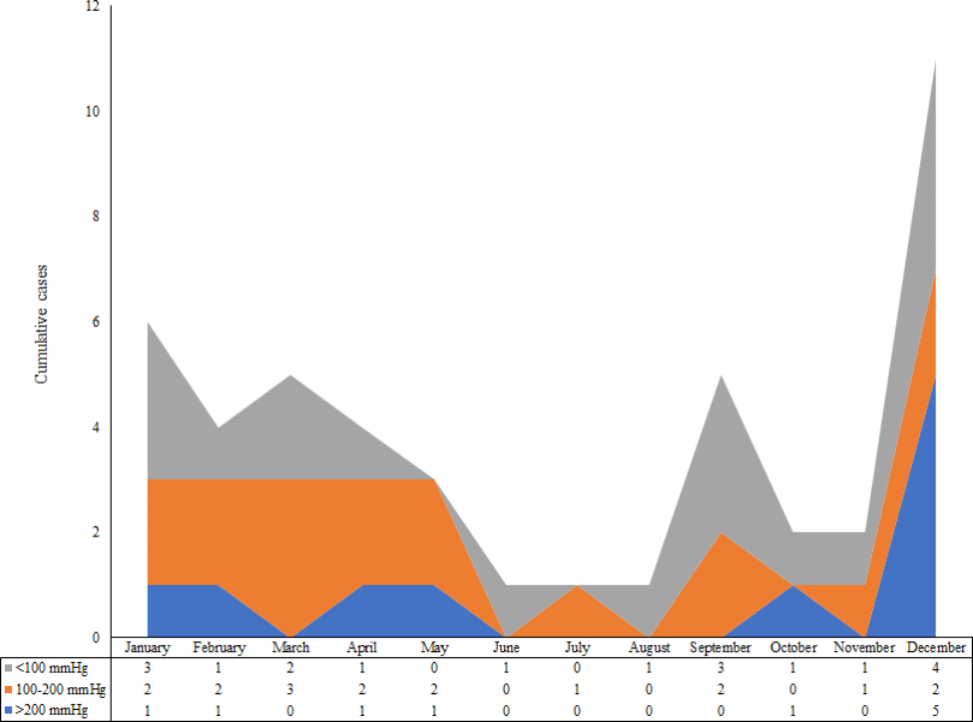
Cumulative number of cases by PaO_2_/FiO_2_ in 12 months FiO_2_, fraction of inspired oxygen; PaO_2_, arterial partial pressure of oxygen

From April 2018 to May 2021, 45 patients with severe CAP and AHRF were diagnosed with psittacosis. *C. psittaci* was detected using mNGS on BALF samples. Droplet digital PCR validation was carried out while preparing this study, all the samples showed positive results of *C. psittaci*. It was found psittacosis occurred throughout the year, especially with the high incidence between September and April. The median PaO_2_/FiO_2_ of the patients was 119.8 (IQR, 73.2 to 183.6) mmHg, and the time distribution did not vary with the PaO_2_/FiO_2_ (Fig. 1). The mean age was 60 ± 14 years, and 27 (60.0%) patients were males. A history of poultry exposure was found in 64.4% of the patients. The median duration from symptom onset to admission was 7 (IQR, 4 to 10) days (Table 1).

**Table 1:** Characteristics of patients with psittacosis-induced severe community-acquired pneumonia and acute hypoxic respiratory failure

	Patients (n = 45)
**Gender (Male)**	27 (60.0)
**Age (y)**	60 ± 14
**Contact history of poultry (n,%)**	29 (64.4)
**Smoking (n,%)**	15 (33.3)
**Symptom onset to admission (days)**	7 (4, 10)
**Symptom onset to ICU (days)**	7 (5, 10)
**BMI (kg/m** ^**2**^ **)**	23.9 ± 2.8
**Underling diseases (n,%)**	
Hypertension	19 (42.2)
Diabetes mellitus	12 (26.7)
Coronary heart disease	8 (17.8)
Chronic heart failure	4 (8.9)
Chronic renal failure	3 (6.7)
Pregnancy	3 (6.7)
Immunosuppression	3 (6.7)
**Complications (n,%)**	
Hepatic injury	24 (53.3)
Cardiac insufficiency	10 (22.2)
Acute renal failure	8 (17.8)
Pleural effusion	8 (17.8)
Altered consciousness	7 (15.6)
Septic shock	3 (6.7)
**Symptom (n,%)**	
Fever	44 (97.8)
Highest temperature (°C)	39.4 (39.0, 40.0)
Cough	36 (80.0)
Expectoration	29 (64.4)
Dyspnea	29 (64.4)
Shortness of breath	17 (37.8)
Chills	13 (28.9)
Fatigue	13 (28.9)
Chest tightness	8 (17.8)
Shivering	7 (15.6)
Muscle soreness	7 (15.6)
Nausea	4 (8.9)
Chest pain	2 (4.4)
**Vital signs**	
Systolic blood pressure (mmHg)	130 ± 25
Diastolic blood pressure (mmHg)	74 ± 12
Heart rate (beats per minute)	100 ± 21
Respiratory rate (times per minute)	24 (20, 30)
APACHE II score	11 (8, 17)
SOFA score	5 (3, 7)

APACHE, Acute Physiology and Chronic Health Evaluation; BMI, body mass index; ICU, intensive care unit; SOFA, sequential organ failure assessment

### Clinical characteristics and laboratory examination

Almost all of the patients had high fever, and the highest recorded temperature was 39.4 °C (IQR, 39.0 to 40.0). Patients commonly presented with cough, expectoration, and dyspnea. The median Acute Physiology and Chronic Health Evaluation (APACHE) II and sequential organ failure assessment (SOFA) scores were 11 (IQR, 8 to 17) and 5 (IQR, 3 to 7), respectively. More than half of the patients (53.3%) had hepatic injury, 10 patients had heart failure, and eight patients had acute renal failure during admission (Table 1).

**Fig. 2:**
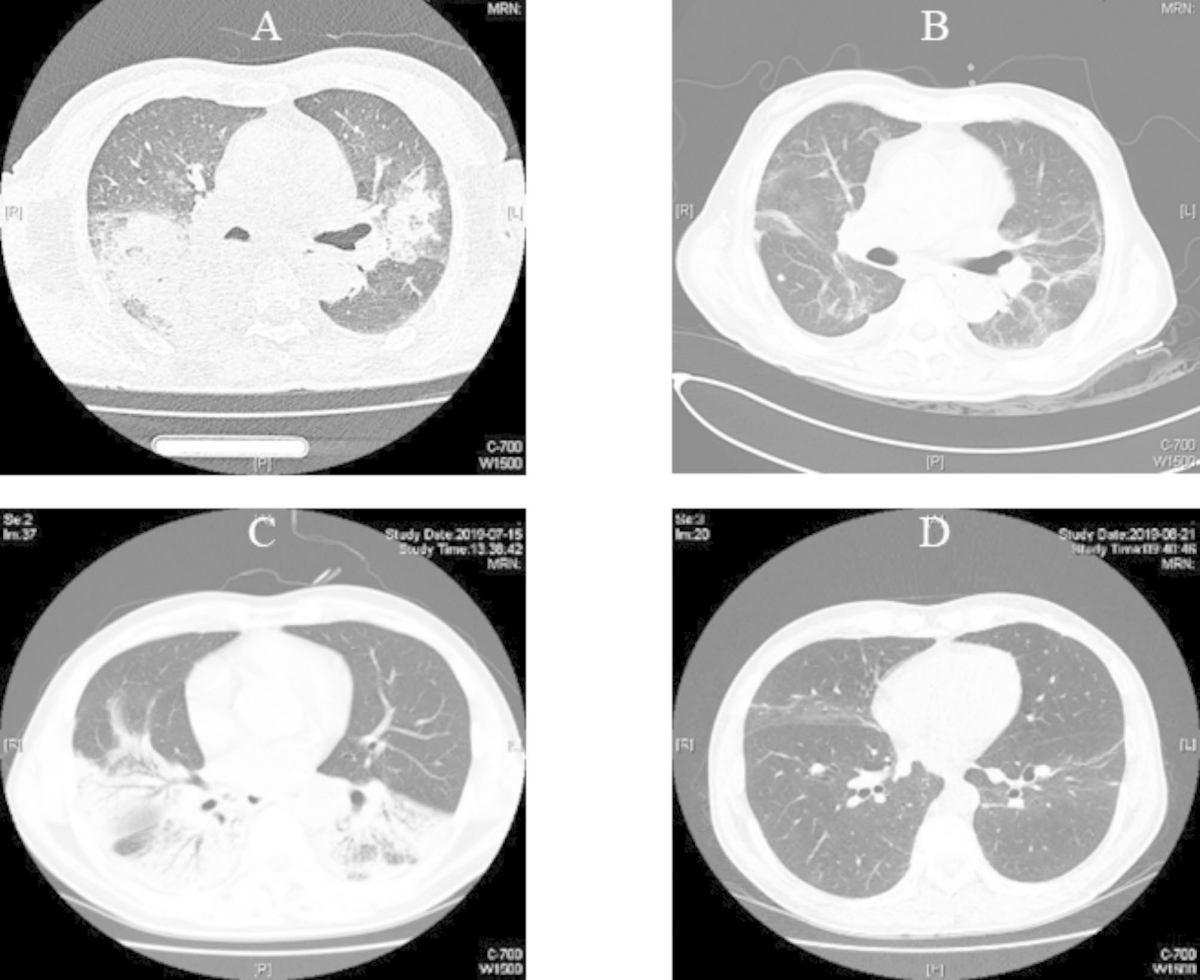
Lung computed tomography findings of the patients with psittacosis caused severe community acquired pneumonia Figure-2**A** was the lung CT of an 80 years old male of psittacosis pneumonia while admission, which showed consolidation and infiltrate in bilateral lungs. Figure-2**B** showed reticular lesions and ground-glass opacities residually in bilateral lungs before discharged from ICU. Figure-2**C** showed a 54 years old female, consolidation and infiltrate in lower lobes of bilateral lungs. Figure-2**D** showed few reticular in bilateral lungs before discharged

The median white blood cell count was 8.39 × 10^9^/L (IQR, 6.02 to 11.77), which was at the upper limit of the normal range (4–10 × 10^9^/L). The median lymphocyte count was 0.50 × 10^9^/L (IQR, 0.34 to 0.58), which was significantly lower than the normal range (0.8 × 10^9^/L; *p* < 0.001). Glutamic-oxaloacetic transaminase, alanine aminotransferase, and bilirubin were mildly elevated. The median serum procalcitonin (PCT) level was 1.64 ng/mL (IQR, 0.42 to 4.88), which was significantly higher than the normal (0.5 ng/mL)[15]. The other laboratory results are shown in Table 2. Lung CT in most patients showed consolidation and infiltrate appeared in multiple lobes and segmental of bilateral lungs, lesions were more common in the lower lobes (Fig. 2).

**Table 2:** Laboratory tests during admission

	Patients (n = 45)
**Blood routine test**	
White blood cell count (× 10^9^/L)	8.39 (6.02, 11.77)
Neutrophil count (× 10^9^/L)	7.30 (5.26, 10.59)
Lymphocyte count (× 10^9^/L)	0.50 (0.34, 0.68)
Monocyte count (× 10^9^/L)	0.29 (0.11, 0.38)
Hemoglobin (g/L)	114.5 (97.0, 128.5)
Hematocrit (%)	32.9 (28.9, 37.5)
Platelets (× 10^9^/L)	200.5 (129.0, 300.0)
**Coagulation function**	
Prothrombin time (s)	13.2 (12.0, 14.7)
Activated partial thromboplastin time (s)	31.2 (28.3, 34.4)
Fibrinogen (mg/dL)	687.5 (465.4, 816.0)
D-Dimer (pg/mL)	3.1 (2.2, 6.8)
**Biochemical tests**	
Albumin (g/L)	27.3 (23.1, 30.6)
Troponin I (ng/mL)	0.02 (0.01, 0.08)
Aspartate aminotransferase (U/L)	64.5 (32.7, 108.0)
Alanine aminotransferase (U/L)	52.6 (37.0, 87.0)
Lactate dehydrogenase (U/L)	485 (399, 644)
Total bilirubin (µmol/L)	13.2 (7.1, 19.5)
Direct bilirubin (µmol/L)	7.0 (5.4, 11.4)
Serum urea nitrogen (mmol/L)	6.2 (4.9, 11.4)
Creatinine (µmol/L)	63.8 (48.0, 82.0)
Glycosylated hemoglobin (%)	6.2 (5.7, 7.0)
B-type brain natriuretic peptide (pg/ml)	178.0 (57.5, 246.5)
**Immunity and infection markers**	
Erythrocyte sedimentation rate (mm/h)	50 (45, 56)
C reactive protein (mg/dL)	24.5 (16.0, 29.3)
CD3 + lymphocyte (/µL)	291 (266, 477)
CD4 + CD3 + lymphocyte (/µL)	195 (172, 368)
CD8 + CD3 + lymphocyte (/µL)	75 (66, 107)
CD4/CD8	2.8 (2.6, 3.0)
Immunoglobulin G (mg/dL)	854.5 (679.5, 1145.8)
Immunoglobulin A (mg/dL)	220.9 (168.5, 281.5)
Immunoglobulin M (mg/dL)	76.4 (51.8, 90.2)
Complement c3 (mg/dL)	90.8 (69.2, 115.0)
Complement c4 (mg/dL)	22.1 (16.8, 29.2)
Procalcitonin (ng/mL)	1.64 (0.42, 4.88)
**Arterial blood gas analysis**	
pH	7.47 (7.41, 7.51)
PaCO_2_ (mmHg)	32.6 (26.5, 38.4)
PaO_2_ (mmHg)	65.5 (59.1, 75.0)
Lactate (mmol/L)	1.4 (1.2, 2.0)
PaO_2_/FiO_2_ (mmHg)	119.8 (73.2, 183.6)

FiO_2_, fraction of inspired oxygen; PaCO_2_, arterial carbon dioxide partial pressure; PaO_2_, arterial partial pressure of oxygen

### Treatment and outcome

Before the diagnosis was confirmed, 30 patients received fluoroquinolone and 2 received azithromycin. None of the patients received tetracycline. The remaining 13 were empirically given β-lactam antibiotics or antivirals included oseltamivir. After the diagnosis was confirmed, 12 patients who initially received fluoroquinolones were shifted to tetracycline, and the other 18 patients continued fluoroquinolones. Among patients who empirically used β-lactam antibiotics or antivirals, eight were given fluoroquinolones combined with tetracycline, two were given tetracycline, and one was given fluoroquinolones alone (Table 2). The median duration from admission to start of targeted therapy was 5 days (IQR, 3 to 10).

Sixteen patients received non-invasive positive pressure ventilation (NIPPV), and four of them were intubated because of failure of NIPPV. Twenty patients (44.4%) were intubated and received invasive mechanical ventilation (IMV), whereas two received veno-venous extracorporeal membrane oxygenation. Four of 45 patients (8.9%) died in the ICU, and the median ICU stay duration was 12 days (IQR, 8 to 21). The median hospitalization duration was 15 days (IQR, 12 to 27) (Table 3). The lung CT of the survival patients before discharge from ICUs most commonly showed consolidation and infiltrates absorbed and residual ground-glass opacities and reticular/fibrotic lesions (Fig. 2). The median CT score at the time of discharged was 4.3 ± 3.1, which was marked decreased compared with admission of 12.5 ± 5.6.

**Table 3:** Treatment and outcome of patients with psittacosis

	Patients (n = 45)
**Initial antibiotic treatment (n,%)**	
β-lactam antibiotics or antivirals	13 (28.9)
Fluoroquinolones	30 (66.7)
Azithromycin	2 (4.4)
**Targeted therapy (n,%)**	
None	2 (4.4)
Fluoroquinolone initially, changed to tetracycline	12 (26.7)
Fluoroquinolone initially, continued after diagnosis	18 (40.0)
Azithromycin initially, continued after diagnosis	1 (2.2)
Azithromycin initially, changed to tetracycline	1 (2.2)
Fluoroquinolone combined with tetracycline after diagnosis	8 (17.8)
Fluoroquinolone after diagnosis	1 (2.2)
Tetracycline after diagnosis	2 (4.4)
Corticosteroid	11 (24.4)
Cumulative dose (mg)	530 (200, 760)
Time from admission (d)	4 (2, 7)
Duration (day)	6 (5, 18)
**Respiratory support**	
HFNC	26 (57.8)
Highest FiO_2_	0.6 (0.5, 0.7)
Highest flow rate (L/min)	50 (40, 60)
NIPPV	16 (35.6)
Highest FiO_2_	0.6 (0.5, 0.8)
Highest PEEP (cmH_2_O)	6 (5, 8)
Failure (n,%)	4 (25.0)
IMV	20 (44.4)
Highest FiO_2_	1.0 (0,8, 1.0)
Highest PEEP (cmH_2_O)	12 (10, 14)
Highest Pi (cmH_2_O)	15 (13, 18)
Duration (hr)	168 (131, 252)
Prone position (n,%)	11 (24.4)
ECMO (n,%)	2 (4.4)
CRRT (n,%)	6 (13.3)
ICU duration (day)	12 (8, 21)
Hospitalization duration (day)	15 (12, 27)
Mortality rate (n,%)	4 (8.9%)

CRRT, continuous renal replacement therapy; ECMO, extracorporeal membrane oxygenation; FiO_2_, fraction of inspired oxygen; HFNC, high-flow nasal canula oxygen therapy; ICU, intensive care unit; IMV, invasive mechanical ventilation; NIPPV, non-invasive positive pressure ventilation; PEEP, positive end expiratory pressure; Pi, inspiratory pressure

### Comparison of treatment methods

Because only a few patients received azithromycin and tetracycline alone, we compared the clinical characteristics and outcomes between patients who received fluoroquinolone initially and shift to tetracycline after diagnosis, fluoroquinolone initially and continued after diagnosis, and fluoroquinolone combined with tetracycline.

There were no significant differences between the three groups in terms of the demographic data, duration from symptom onset to admission, and disease severity (Table 4). The white blood cell and neutrophil counts were significantly higher among patients who received fluoroquinolone initially and after diagnosis compared to the other patients (*p* = 0.015). Among patients who received fluoroquinolone initially and after diagnosis, 11 received IMV and 6 underwent continuous renal replacement therapy, which was more common compared to the other two groups. There was no difference in in-hospital mortality and hospitalization duration between the three groups.

**Table 4:** Comparison between treatment methods

	Fluoroquinolone initially, continued after diagnosis (n = 18)	Fluoroquinolone initially, changed to tetracycline (n = 12)		Fluoroquinolone combined with tetracycline after diagnosis (n = 8)	*P*-value
Age (y)	56 (47, 64)	62 (52, 68)		61 (54, 73)	0.757
Gender (Male)	18 (55.6)	10 (83.3)		2 (25.0)	0.034
Symptom onset to admission (days)	9 (5, 10)	7 (6, 7)		4 (4, 7)	0.140
Symptom onset to ICU (day)	9 (5, 11)	7 (6, 9)		10 (5, 10)	0.556
APACHE II score	11 (8, 20)	7 (6, 13)		14 (10, 15)	0.901
SOFA score	5 (2, 10)	3 (2, 4)		6 (4, 6)	0.416
White blood cell count (× 10^9^/L)	10.32 (7.36, 11.80)	7.58 (5.00, 11.98)		6.38 (5.70, 7.50)	0.015
Neutrophil count (× 10^9^/L)	9.47 (6.89, 11.04)	6.77 (3.94, 10.03)		5.73 (4.94, 6.82)	0.015
Lymphocyte count (× 10^9^/L)	0.52 (0.36, 0.67)	0.62 (0.42, 0.86)		0.46 (0.33, 0.54)	0.659
Aspartate aminotransferase (U/L)	80.0 (63.0, 123.0)	96.0 (58.0, 198.8)		52.1 (40.0, 74.0)	0.024
Alanine aminotransferase (U/L)	43.5 (36.0, 69.0)	75.0 (46.4, 121.0)		70.0 (59.0, 87.0)	0.082
Serum urea nitrogen (mmoL/l)	6.58 (4.89, 7.43)	5.10 (3.83, 9.40)		6.49 (2.80, 7.51)	0.329
Creatinine (µmol/L)	68.4 (44.5, 108.1)	71.5 (52.6, 80.0)		53.3 (33.8, 73.6)	1.000
PaO_2_/FiO_2_ (mmHg)	120.8 (67.8, 200.0)	100.0 (92.5, 119.6)		133.6 (80.0, 148.3)	0.329
Duration from admission to targeted therapy	-	-		6 (2, 10)	-
Respiratory support					
HFNC	13 (72.2)	6 (50.0)		4 (50.0)	0.376
NIPPV	7 (38.9)	3 (25.0)		4 (50.0)	0.509
NIPPV failure	3 (16.7)	1 (8.3)		0 (0)	0.423
IMV	11 (61.1)	5 (41.7)		1 (12.5)	0.069
CRRT	4 (22.2)	0 (0)		0 (0)	0.083
Corticosteroids	6 (31.6)	1 (7.7)		2 (28.6)	0.269
Mortality rate	1 (5.6)	2 (16.7)		0 (0)	0.352
ICU duration (d)	14 (12, 22)	11 (9, 19)		7 (6, 11)	0.117
Hospitalization duration (d)	15 (13, 27)	14 (12, 26)		17 (14, 24)	0.649

APACHE, Acute Physiology and Chronic Health Evaluation; CRRT, continuous renal replacement therapy; FiO_2_, fraction of inspired oxygen; HFNC, high-flow nasal canula oxygen therapy; ICU, intensive care unit; IMV, invasive mechanical ventilation; NIPPV, non-invasive positive pressure ventilation; PaO_2_, arterial partial pressure of oxygen, SOFA, sequential organ failure assessment.

## Discussion

To the best of our knowledge, this was the largest cohort study of psittacosis accompanied by AHRF from mainland China. In this study, we found psittacosis may present with varying AHRF severity, and the common seasons of psittacosis were autumn and winter. Fluoroquinolones may have efficacy equivalent to tetracycline. Considering the limited diagnostic ability, standard empirical treatment should follow CAP guidelines [12, 16], which recommend that treatment should cover atypical pathogens to improve the prognosis of patients with psittacosis and AHRF.

*C. psittaci* was reported to account for 2.3% of cases of severe CAP [3]. The mortality rate of patients with psittacosis admitted to the ICU was as high as 15%[17]. It is essential to recognize psittacosis based on the symptoms in patients with CAP and AHRF. Patients should receive early targeted therapy for a better prognosis. *C. psittaci* infection presents with an abrupt onset of fever, chills, headache, malaise, and myalgias. A non-productive cough is usually present and can be accompanied by breathing difficulty or chest tightness. Radiographic findings may include lobar or interstitial infiltrates [1]. About 70% of psittacosis patients had a known past exposure to poultry [18]. The incubation period for the illness is 5–14 days [19]. Age older than 65 years, male sex [20], and abnormal CK and BNP levels [21] were the risk factors for severe cases.

Similar to previous reports, there were atypical clinical manifestations of psittacosis in our study, which makes differentiation of psittacosis from other infections difficult. Although psittacosis can occur at any time of the year, most patients presented in autumn and winter. Therefore, the outbreak of psittacosis coincided with that of influenza. However, most psittacosis patients had a history of poultry exposure, and it is important to differentiate it from other infections that are transmitted from poultry, including H7N9, H5N1, H5N6, etc. The difficulty in differentiation between these pathogens partly explains why 30% of the patients in this study received antivirals initially. In addition, we found the white blood cell count was in the normal range, the lymphocyte count was slightly reduced, and PCT was slightly elevated (almost lower than 2 ng/mL). Most patients with psittacosis had impaired liver function. Lung CT showed consolidation and infiltrates appeared in multiple lobes and segments of bilateral lungs. These presentations may help to differentiate psittacosis from other similar infections that present with severe CAP.

Rapid diagnosis is essential for a good prognosis of CAP patients. Psittacosis can be diagnosed on the basis of a suggestive clinical presentation and detection of *C. psittaci* in human specimens. The current confirmatory laboratory tests for psittacosis include PCR and serologic tests (complement binding reactions, enzyme-linked immunosorbent assay, immunofluorescence tests, and immuno-peroxidase tests)[22]. In recent years, PCR has become the most commonly used diagnostic method for psittacosis [2, 23]. However, to ensure biosafety, this test can only be performed in special laboratories [24].

mNGS can theoretically detect all pathogens in a clinical sample and is especially suitable for rare, novel, and atypical etiologies of complicated infectious diseases [25]. The use of NGS for the diagnosis of a suspected outbreak of psittacosis-induced severe CAP and ARDS was first reported in 2014 [9]. With the recent increase in the use of mNGS, psittacosis is being increasingly and rapidly screened and diagnosed. It seemed superior to the traditional methods [10, 11]. In particular, mNGS has great potential for use for rapid diagnosis during public health crisis [26, 27]. Rapid diagnosis is essential for critically ill patients with severe pneumonia and AHRF. In our previous work, we found that mNGS improved the sensitivity of pathogen detection in BALF and provides clinical guidance for management. Additionally, dynamic changes in reads could indirectly reflect therapeutic effectiveness [28]. Due to its sensitivity, speed, and cost-effectiveness, mNGS has the potential for routine use in diagnostics, and may even partly replace the traditional paradigm of serial tests [25].

In this study, psittacosis patients with AHRF who were admitted to the ICU had a better outcome, with a mortality rate of 8.9%, which was lower than previous reports [17]. In addition to appropriate organ support, standard empirical treatment for CAP covered atypical pathogens, including *C. psittaci*, which might play an important role. Tetracyclines are the drugs of choice for *C. psittaci* infection in humans, and are prescribed for 10–14 days [1, 12, 16]. Most *C. psittaci* infections respond to antibiotics within 1–2 days. In this study, 30 patients received fluoroquinolones as the initial empirical antibiotics. After diagnosis, only 12 patients were shifted to tetracycline, and the other patients continued using fluoroquinolones. Because few patients had received azithromycin or tetracycline individually, we only compared patients who received fluoroquinolone individually with those who received fluoroquinolone combined with tetracycline. Although the proportion of patients who required IMV and continuous renal replacement therapy was slightly greater among the fluoroquinolone group compared to the other groups, the mortality rate and hospitalization stay duration were not significantly different between the groups. Although tetracycline is the preferred antibiotic for chlamydial infections in non-pregnant adults, some studies have reported that fluoroquinolones are active against chlamydia in vitro or in veterinary medicine [29, 30]. The results of this study provide more evidence for the use of fluoroquinolone for patients with psittacosis, especially those with severe CAP and AHRF.

There were still some limitations to this study. First, this was a retrospective study with a small sample size, and the patients were exclusively from mainland China. Therefore, the results may not be generalizable to all populations. However, considering the low incidence rate of psittacosis, the results of this study have certain clinical value. Second, we could not include all severe CAP patients treated at the study centers. Therefore, the actual incidence of psittacosis among severe CAP patients could not be calculated. Third, microbiological diagnosis of psittacosis was based on mNGS, which is not included in the diagnostic criteria for psittacosis. However, droplet digital PCR validation (DDPCR) was carried out in this study, which could strengthen the diagnose accuracy. Fourth, because of insufficient data on respiratory support, we could not analyze respiratory mechanics parameters or respiratory support parameters in patients with AHRF. Lastly, only four patients died in this study. Therefore, factors related to poor prognosis could not be determined.

## Conclusion

Psittacosis caused severe CAP was not rare, especially in the patients with the history of exposure to poultry or birds. It may present with varying AHRF severity. Novel microbiological technologies may improve the diagnostic potential. Empirical treatment that covers atypical pathogens may benefit such patients, which fluoroquinolones might be considered as an alternative. The results need to be verified in large, well-designed, prospective randomized controlled studies to further evaluate the treatment and outcomes of psittacosis.

## Data Availability

Data is deposited in China National Microbiology Data Center (NMDC) with accession numbers NMDC10018302 (https://nmdc.cn/resource/genomics/project/detail/NMDC10018302).

## List of abbreviations

AHRF: Acute hypoxic respiratory failure
APACHE: Acute Physiology and Chronic Health Evaluation
CAP: Community-acquired pneumonia
CT: Computed tomography
DDPCR: Droplet digital PCR validation
ICU: Intensive care units
IMV: Invasive mechanical ventilation
IQR: Interquartile range
mNGS: Metagenomic next-generation sequencing
NIPPV: Non-invasive positive pressure ventilation
PaO_2_: Partial oxygen pressure
PCR: Polymerase chain reaction
PCT: Procalcitonin
SD: Standard deviation
SOFA: Sequential organ failure assessment
