# Publisher Correction: Psittacosis caused severe community-acquired pneumonia accompanied by acute hypoxic respiratory failure: a multicenter retrospective cohort study from China

**DOI:** 10.1186/s12879-023-08543-y

**Published:** 2024-01-15

**Authors:** Xiao Tang, Na Wang, Gang Liu, Hai Tan, Ai-Min Li, Yan-Qiu Gao, Meng-Ying Yao, Na Wang, Hui-Dan Jing, Qing-Guo Di, Liang Chen, Rui Wang, Xu-Yan Li, Ying Li, Xue Yuan, Yu Zhao, Qi Li, Zhao-Hui Tong, Bing Sun

**Affiliations:** 1 Department of Respiratory and Critical Care Medicine, Beijing Institute of Respiratory Medicine and Beijing Chao-Yang Hospital, Capital Medical University, Beijing, China; 2 Department of Pulmonary and Critical Care Medicine, Beijing Luhe Hospital, Capital Medical University, Beijing, China; 3 Department Pulmonary and Critical Care Medical Center, Xinqiao Hospital, Army Medical University, the Chinese People’s Liberation Army Respiratory Disease Institute, Chongqing, China; 4 Department of Respiratory and Critical Care Medicine, General Hospital of Ningxia Medical University, Ningxia Hui Autonomous Region, Xi Ning, China; 5 Respiratory and Critical Care Medicine, First Hospital of Shanxi Medical University, Taiyuan, Shanxi Province China; 6 Respiratory Intensive Care Unit, Zhengzhou Central Hospital Affiliated to Zhengzhou University, Zhengzhou, Henan Province China; 7 Department of Respiratory Intensive Care Unit, The First Affiliated Hospital of Zhengzhou University, Zhengzhou, Henan Province China; 8 Department of Pulmonary, The First Hospital of Fangshan District, Beijing, China; 9 Department of Intensive Care Unit, Daping Hospital, Army Medical University, Chongqing, China; 10 Department of Pulmonary and Critical Care Medicine, Cangzhou Central Hospital, Cangzhou, Hebei Province China; 11 Department of Respiratory and Critical Care Medicine, Beijing Jingmei Group General Hospital, Beijing, China

**Publisher Correction: BMC Infect Dis 23, 532 (2023)**

**https://doi.org/10.1186/s12879-023-08283-z**

The original publication of this article [1] contained 2 errors as a result of the publication process:

- An incorrect affiliation was listed for Na Wang
- One author (Na Wang) was missing

The original publication has been updated. The publisher apologizes for the inconvenience caused to the authors & readers.
